# Sphingosine Facilitates SNARE Complex Assembly and Activates Synaptic Vesicle Exocytosis

**DOI:** 10.1016/j.neuron.2009.04.024

**Published:** 2009-06-11

**Authors:** Frédéric Darios, Catherine Wasser, Anastasia Shakirzyanova, Artur Giniatullin, Kerry Goodman, Jose L. Munoz-Bravo, Jesica Raingo, Jernej Jorgačevski, Marko Kreft, Robert Zorec, Juliana M. Rosa, Luis Gandia, Luis M. Gutiérrez, Thomas Binz, Rashid Giniatullin, Ege T. Kavalali, Bazbek Davletov

**Affiliations:** 1 MRC Laboratory of Molecular Biology, Cambridge CB2 0QH, UK; 2 Department of Neuroscience, UT Southwestern Medical Center, Dallas, TX 75390, USA; 3 Institute of Biochemistry and Biophysics KSC RAS, Kazan 420503, Russia; 4 State Medical University, Kazan 420012, Russia; 5 Laboratory of Neuroendocrinology and Molecular Cell Physiology, University of Ljubljana, 1000 Ljubljana, Slovenia; 6 Celica Biomedical Center, 1000 Ljubljana, Slovenia; 7 Teófilo Hernando Institute and Department of pharmacology and therapeutics, Autonomous University of Madrid, 28049 Madrid, Spain; 8 Institute of Neurosciences, CSIC-Miguel Hernández University, 03550 Alicante, Spain; 9 Department of Biochemistry, Medizinische Hochschule Hannover, 30625 Hanover, Germany; 10 Virtanen Institute for Molecular Sciences, University of Kuopio, 70600 Kuopio, Finland

**Keywords:** MOLNEURO, CELLBIO, SIGNALING

## Abstract

Synaptic vesicles loaded with neurotransmitters fuse with the plasma membrane to release their content into the extracellular space, thereby allowing neuronal communication. The membrane fusion process is mediated by a conserved set of SNARE proteins: vesicular synaptobrevin and plasma membrane syntaxin and SNAP-25. Recent data suggest that the fusion process may be subject to regulation by local lipid metabolism. Here, we have performed a screen of lipid compounds to identify positive regulators of vesicular synaptobrevin. We show that sphingosine, a releasable backbone of sphingolipids, activates synaptobrevin in synaptic vesicles to form the SNARE complex implicated in membrane fusion. Consistent with the role of synaptobrevin in vesicle fusion, sphingosine upregulated exocytosis in isolated nerve terminals, neuromuscular junctions, neuroendocrine cells and hippocampal neurons, but not in neurons obtained from *synaptobrevin-2* knockout mice. Further mechanistic insights suggest that sphingosine acts on the synaptobrevin/phospholipid interface, defining a novel function for this important lipid regulator.

## Introduction

Neurotransmission occurs when synaptic vesicles loaded with neurotransmitters fuse with the plasma membrane causing release of their content. This neuronal exocytosis requires three SNARE (soluble-N-ethylmaleimide sensitive factor attachment protein receptor) proteins: synaptobrevin-2 (also known as VAMP-2) on the synaptic vesicle, and syntaxin-1 with SNAP-25 on the plasma membrane (Jahn and Scheller, 2006; Sollner, 2003; Sudhof and Rothman, 2009). Formation of the SNARE ternary complex by the three proteins is an essential step toward membrane fusion.

It is critical for neuronal physiology that synaptic vesicle exocytosis takes place with high spatial and temporal precision. Preventing uncontrolled formation of SNARE complex can be an important point for regulation of vesicle fusion and thereby of neurotransmission. Interestingly, synaptobrevin in synaptic vesicles does not readily react with syntaxin and SNAP-25 (Hu et al., 2002). Two alternative mechanisms could account for constitutive downregulation of synaptobrevin activity: synaptophysin, a small synaptic vesicle marker, can interact with synaptobrevin's transmembrane part (Edelmann et al., 1995; Yelamanchili et al., 2005) and the cytoplasmic part of synaptobrevin has been shown to interact with lipid membranes (Caccin et al., 2003; Quetglas et al., 2000). Recent studies highlighted a likely role of lipid metabolism in regulation of vesicle fusion (Bankaitis and Morris, 2003; Fivaz and Meyer, 2003; Humeau et al., 2001; Rohrbough and Broadie, 2005; Sollner, 2007; Wenk and De Camilli, 2004). Since vesicular properties of synaptobrevin must be essential in SNARE-mediated membrane fusion, we decided to screen a library of lipid compounds using isolated synaptic vesicles to gain insight into possible regulators of synaptobrevin function. We now show that sphingosine, an essential releasable constituent of cell membranes (Hannun and Obeid, 2008; Lahiri and Futerman, 2007; Posse de Chaves, 2006), can potently activate synaptobrevin and also enhance SNARE-mediated synaptic vesicle exocytosis.

## Results

### Identification of Sphingosine as an Activator of Vesicular Synaptobrevin

**Figure 1:**
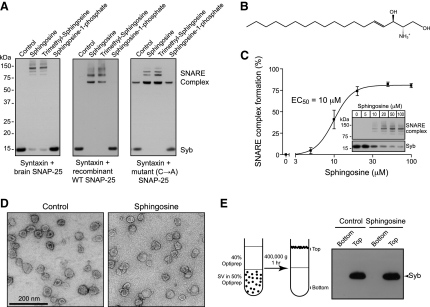
Sphingosine Activates Synaptobrevin in Synaptic Vesicles for SNARE Assembly (A) Rat brain synaptic vesicles were mixed with syntaxin/SNAP-25 made with syntaxin 1A (aa 1–261) and either brain-purified, recombinant wild-type (WT), or recombinant SNAP-25 devoid of cysteines (SNAP-25 C→A) in the presence of indicated lipids. Following 30 min incubation at 22°C the reactions were analyzed by SDS-PAGE and western immunoblotting using an anti-synaptobrevin antibody. Note mass transition of monomeric synaptobrevin into the SNARE complex in the case of sphingosine and its derivative trimethyl-sphingosine. (B) Chemical structure of sphingosine, a backbone lipid of cellular membranes. (C) Titration of sphingosine indicates EC_50_ of 10 μM. Inset, representative immunoblot of synaptic vesicles showing sphingosine-dependent transition of monomeric synaptobrevin into the SNARE complex. Error bars represent SEM, n = 7. (D) Negative stain electron microscopy images demonstrating that 50 μM sphingosine does not affect overall morphology of synaptic vesicles. (E) Left panel shows a schematic outline of the flotation experiment. Immunoblot (right panel) shows that synaptobrevin cofloats with synaptic vesicles to the top of the discontinuous Optiprep gradient following treatment with 50 μM sphingosine. Syb, synaptobrevin.

For screening of lipids with a potential role in exocytosis, we purified synaptic vesicles from rat brain by a flotation procedure (Hu et al., 2002) and incubated them with preassembled soluble syntaxin1/SNAP-25 heterodimers in the presence of various lipid compounds (Table 1) for 30 min at 22°C. A hallmark of the neuronal SNARE complex is its resistance to dissociation by chaotropic agents and detergents, even SDS (Hu et al., 2002). Therefore synaptobrevin's ability to engage syntaxin/SNAP-25 can be analyzed through the formation of SDS-resistant SNARE complexes. This analysis was done by Western immunoblotting using a monoclonal antibody which detects both the free and engaged forms of synaptobrevin. Lipids were regarded as synaptobrevin activators if, at 50 μM concentration, they could promote formation of the SNARE complexes, at the same time decreasing the amount of monomeric synaptobrevin (Figure 1A). Remarkably, among the molecules tested, only sphingosine and some of its derivatives were able to stimulate synaptobrevin in synaptic vesicles (Table 1 and Figure 1A). We tested synaptobrevin availability using the soluble part of syntaxin (aa 1–261) preassembled with either brain SNAP-25 (Hu et al., 2002), wild-type recombinant SNAP-25 or recombinant SNAP-25 in which four cysteines were mutated (Siddiqui et al., 2007). Sphingosine activated synaptobrevin regardless of which SNAP-25 was used (Figure 1A). No synaptobrevin-containing assemblies were observed if syntaxin and/or SNAP-25 were omitted (data not shown). Since sphingosine (Figure 1B) is an essential constituent of neuronal membranes, which can be enzymatically released into the cytosol, and its levels significantly change in brain pathologies and with aging (Colombaioni and Garcia-Gil, 2004; Dasgupta et al., 2007; He et al., 2008), we decided to investigate the sphingosine effect in detail.

Titration experiments demonstrated that sphingosine allows synaptobrevin to form ternary SNARE complexes in a dose-dependent manner, with an EC_50_ ∼10 μM (Figure 1C). Although micromolar sphingosine concentrations are used in cellular experiments, it was important to rule out a possibility of sphingosine compromising the integrity of synaptic vesicle membranes. We therefore employed two approaches. First, synaptic vesicles were incubated with or without 50 μM sphingosine and the reactions were analyzed by negative-stain electron microscopy. Figure 1D shows that synaptic vesicle membranes appear intact after one hour incubation in the presence of 50 μM sphingosine. Second, we employed a flotation assay where synaptic vesicles, with or without sphingosine, were floated on a discontinuous Optiprep gradient. The presence of synaptobrevin in the samples was probed by immunoblotting. Figure 1E shows that synaptobrevin remains firmly associated with synaptic vesicles (*top* fraction) following the sphingosine treatment.

### Sphingosine Activates Vesicle Exocytosis

**Figure 2:**
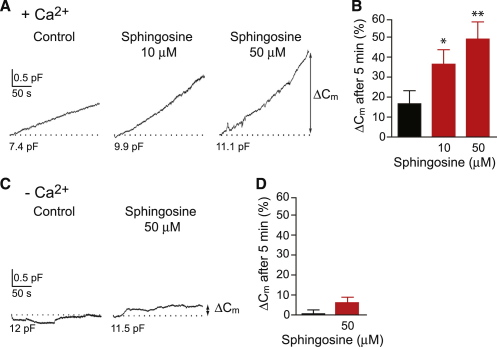
Sphingosine Dialyzed into Rat Pituitary Melanotrophs Enhances Exocytosis (A) Graphs showing calcium-induced changes in membrane capacitance (C_m_) measured by the patch-clamp technique. Sphingosine augments the increase in C_m_. Dashed lines denote resting C_m_ values. (B) The difference in C_m_ (ΔC_m_) was measured 300 s following the establishment of the whole-cell recording as indicated in (A). Mean ΔC_m_ expressed as percentage relative to the resting C_m_. Dialyzed sphingosine increased amplitudes 2-fold at 10 μM (n = 15 cells; ^∗^p ≤ 0.05) and 3-fold at 50 μM (n = 15 cells; ^∗∗^p < 0.01) relative to the control (n = 16 cells). (C) Graphs showing changes in C_m_ in the absence of stimulating calcium. Dialysis of sphingosine at 50 μM into the cytosol led to a small increase in C_m_. (D) Bar chart showing that sphingosine does not significantly affect ΔC_m_ measured as indicated in (C) (control: n = 10; 50 μM sphingosine: n = 15; p > 0.1). The Student's t test was used for pairwise comparisons. In the bar charts, error bars represent SEM.

To test whether sphingosine can activate exocytosis we used a whole-cell patch-clamp setup. Sphingosine was dialyzed into the cytosol of cultured rat pituitary intermediate lobe cells (melanotrophs), which express synaptobrevin-2 (Jacobsson and Meister, 1996). We then measured membrane capacitance (C_m_), a parameter linearly related to the plasma membrane area, which increases upon exocytosis (Neher and Marty, 1982). The cytosol dialysis with 1 μM Ca^2+^ resulted in an increase in C_m_ of 16.7% ± 6.5%. Inclusion of 10 μM sphingosine into the patch pipette solution doubled the average Ca^2+^-dependent increase in C_m_ (36.5% ± 7.2%; Figures 2A and 2B). With 50 μM sphingosine in the pipette solution, the increase in C_m_ was 49% ± 8.6% (Figures 2A and 2B). In the absence of stimulating calcium in the pipette, cytosol dialysis of sphingosine (50 μM) did not significantly affect C_m_ (Figures 2C and 2D). The patch-clamp capacitance experiments were also performed with bovine chromaffin cells but in this case exocytosis was triggered by electrical stimulation. Two minutes after establishing the whole cell configuration, 200 ms depolarizing pulses were applied at 1 min intervals. Dialyzed sphingosine at 10 μM and 50 μM concentrations enhanced the secretory response in chromaffin cells ∼1.2- and 1.6-fold, respectively (see Figure S1 available online). In the absence of stimulation, addition of sphingosine did not significantly change resting membrane capacitance (control: 8.5 ± 0.5 pF; 10 μM sphingosine: 7.7 ± 0.4 pF; 50 μM sphingosine: 7.8 ± 0.4 pF). Together, these results indicate that sphingosine-mediated increase in the plasma membrane area is likely due to enhanced vesicle exocytosis rather than simple incorporation of the lipid into the membrane.

**Figure 3:**
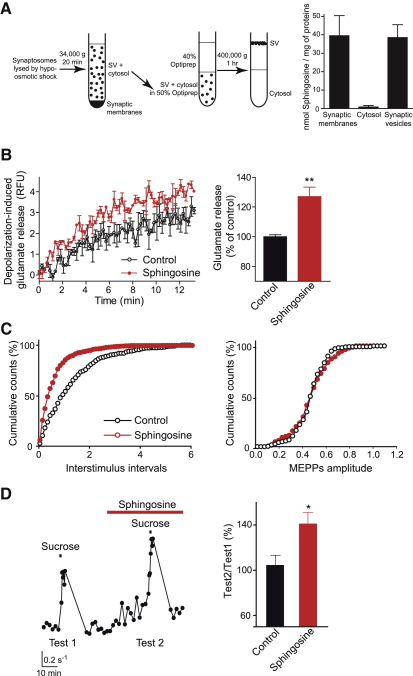
Sphingosine Incorporates into Synaptic Vesicles and Stimulates Vesicle Exocytosis in Synaptosomes and Neuromuscular Junctions (A) Radioactive sphingosine (50 μM) added to rat brain synaptosomes can reach synaptic vesicles. Left panel, schematic describing the procedure for isolation of synaptic fractions. Right panel, bar chart showing that externally added sphingosine incorporates into both synaptic membranes and synaptic vesicles whereas only small amounts of sphingosine are present in the cytosol. (B) Left panel, sphingosine treatment (20 μM) enhances potassium-evoked, calcium-dependent glutamate release from isolated synaptosomes. Curves are from an experiment performed in triplicates (paired t test, p < 0.001). Right panel, end-point calcium-dependent release measured after 10 min of stimulation. Sphingosine enhances glutamate release by 27% (Student's t test, n = 9; ^∗∗^p < 0.001). (C) Left panel, cumulative distribution of inter-event intervals of miniature endplate potentials (MEPPs) in a mouse neuromuscular synapse before and after application of 50 μM sphingosine. Right panel, cumulative distribution of MEPP amplitudes before and after application of 50 μM sphingosine. Note that sphingosine increases frequency but does not change the amplitude of MEPPs. (D) Sucrose-evoked quantal release at the neuromuscular junction before and after application of 50 μM sphingosine. Right panel, averaged data of two consecutive sucrose applications (test-1 followed by test-2 in 60 min). Test-2 was performed either in the absence (control, n = 4; p > 0.05) or presence of sphingosine (n = 5; Mann-Whitney test, ^∗^p < 0.05). Data are presented as test-2 to test-1 ratio. In the bar charts, error bars represent SEM.

To test sphingosine action on synaptic vesicle exocytosis, we first investigated the ability of externally added sphingosine, an amphiphilic lipid, to penetrate biological membranes. We incubated rat brain nerve terminals (synaptosomes) with radioactive sphingosine for 10 min and after a wash step disrupted synaptosomes by hypo-osmotic shock. Following separation of synaptic fractions by centrifugation, distribution of sphingosine in synaptic membranes, synaptic cytosol and synaptic vesicles was analyzed by liquid scintillation (Figure 3A). Sphingosine distributed equally between synaptic membranes and vesicles with a small amount being present in cytosol when normalized to the amount of protein. We then probed potassium-stimulated glutamate release from nerve terminals isolated from adult rat brain using a fluorometric assay (McMahon et al., 1992). Pretreatment of synaptosomes with sphingosine for 10 min before addition of potassium significantly augmented glutamate release (Figure 3B). To probe whether the sphingosine effect is indeed linked to exocytosis of small synaptic vesicles, we analyzed miniature endplate potentials (MEPPs) in mouse neuromuscular junctions. Sphingosine increased frequency of MEPPs by ∼30% without changes in their amplitude (Figure 3C). Next, we tested sphingosine action in sucrose-evoked exocytosis reflecting the readily releasable pool of synaptic vesicles (Figure 3D). In control experiments, two transient (1 min long) local applications of 60 mM sucrose to synaptic regions with an interval of 60 min produced equal bursts of quantal events (Figure 3D; test-2/test-1 ratio is 104.1% ± 8.6%). In the presence of 50 μM sphingosine, however, the second peak response was significantly augmented (Figure 3D; test-2/test-1 ratio is 140.7% ± 10.1%) indicating a larger pool of readily releasable vesicles.

### SNARE-Mediated Mechanisms Account for the Sphingosine Action in Exocytosis

**Figure 4:**
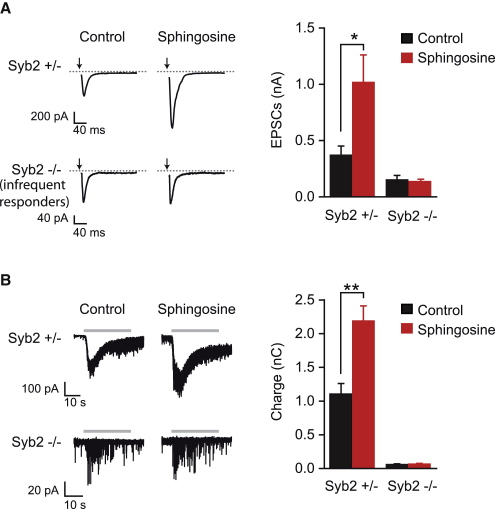
Synaptobrevin-2 Involvement in Sphingosine-Enhanced Transmitter Release (A) Left panel, representative traces of excitatory postsynaptic currents (EPSCs) evoked by field stimulation in *Syb2^+/−^* or *synaptobrevin-2*-deficient (*Syb2^−/−^*) neurons treated with 50 μM sphingosine or vehicle (control). Arrows indicate application of the current. Right panel, bar chart showing a significant increase in the average maximum EPSC amplitudes of sphingosine-treated wild-type neurons compared to nontreated control neurons (control, n = 5; sphingosine, n = 5; ^∗^p < 0.03). No difference in amplitudes was detected in sphingosine-treated compared to nontreated *Syb2^−/−^* neurons (control, n = 5; sphingosine, n = 5, p > 0.72). (B) Left panel, representative traces of hypertonic sucrose-induced (gray bars) transmitter release in *Syb2^+/−^* and *Syb2^−/−^* neurons treated with either 50 μM sphingosine or vehicle. Right panel, bar chart of the charge transfer during the first 10 s of sucrose stimulation showing a 2-fold increase in wild-type (control, n = 11; sphingosine, n = 17; ^∗∗^p < 0.002) and no increase in *Syb2^−/−^* neurons (control, n = 14; sphingosine, n = 17; p > 0.22) after sphingosine addition. In the bar charts, error bars represent SEM. *Syb2*, *synaptobrevin-2*. The Student's t test was used for pairwise comparisons.

To test whether sphingosine action involves synaptobrevin, we probed its effects in a *synaptobrevin* knockout model (*Syb2^−/−^*). Mice deficient in *synaptobrevin-2* die immediately after birth; however, embryonic neurons from these animals survive in culture (Deak et al., 2004; Schoch et al., 2001). Heterozygous mice, on the other hand, are viable and have normal synaptic characteristics. In cultures obtained from heterozygous mice, application of 50 μM sphingosine caused a 2.7-fold increase in the amplitudes of excitatory postsynaptic currents (EPSCs) induced by field stimulation (Figure 4A). *Syb2^−/−^* neurons exhibit a severe reduction in evoked exocytosis and endocytosis especially in response to stimulation at low frequencies (1 Hz). Nevertheless, in some cells one can detect occasional responses to stimulation and sphingosine effects were tested in these responding cells. The responses recorded from *Syb2^−/−^* synapses did not show an increase after sphingosine treatment (Figure 4A). In contrast to field-evoked stimulation, *Syb2^−/−^* neurons reproducibly respond to hypertonic sucrose stimulation albeit at a reduced level (∼6% compared to nontreated wild-type neurons; Schoch et al., 2001). Heterozygous neurons treated with sphingosine had a 2-fold increase in their responses to hypertonic sucrose application compared to neurons from nontreated heterozygous cultures. Sphingosine-treated *Syb2^−/−^* neurons, however, did not exhibit a significant increase in their hypertonic sucrose responses compared with nontreated neurons (Figure 4B). Together these results indicate that synaptobrevin is necessary for the sphingosine-induced increase in vesicle fusion.

To probe the ability of sphingosine to promote SNARE assembly inside neurons, we utilized the fact that botulinum neurotoxins can proteolyze monomeric but not assembled SNAREs (Hayashi et al., 1994). We employed botulinum neurotoxins D and E which can enter neurons and specifically cleave synaptobrevin and SNAP-25, respectively. Hippocampal neurons were incubated in the presence or absence of both sphingosine and neurotoxins for 30 min and then total amounts of monomeric synaptobrevin and SNAP-25 were analyzed by western immunoblotting. Figure S2 shows that application of sphingosine to neurons impeded proteolysis of synaptobrevin and SNAP-25 by the neurotoxins demonstrating upregulation of SNARE assembly.

### Activation of Exocytosis by Endogenous Production of Sphingosine

**Figure 5:**
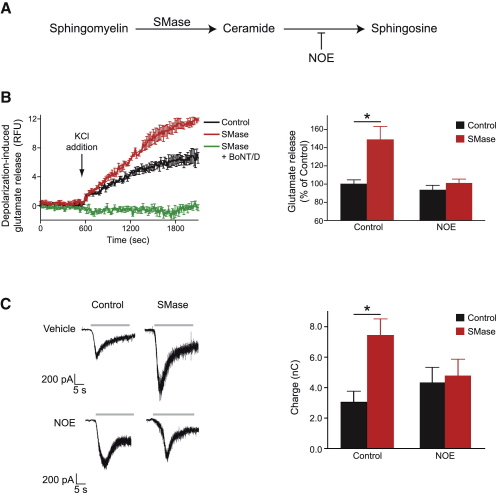
Sphingomyelinase-Enhanced Release of Neurotransmitters Is Blocked by Synaptobrevin-Cleaving Botulinum Neurotoxin D and by a Ceramidase Inhibitor (A) Schematic of enzymatic release of endogenous sphingosine from membrane sphingomyelin. Sphingomyelinase (SMase) initiates hydrolysis of sphingomyelin, while sphingosine is released by ceramidase which can be blocked by N-oleoylethanolamine (NOE). (B) Left panel, SMase treatment (1 unit/ml) enhances KCl-evoked, calcium-dependent glutamate release from synaptosomes. Botulinum toxin D (BoNT/D, 6 nM) potently blocks SMase-enhanced glutamate release. Right panel, bar graph showing normalized glutamate release following 10 min stimulation by KCl. SMase (1 unit/ml) and ceramidase inhibitor, NOE (150 μM) were added 15 and 30 min before addition of KCl, respectively (n = 5 ^∗^p < 0.05). (C) Left panel, representative traces of hypertonic sucrose-induced (gray bars) neurotransmitter release in rat hippocampal cultured neurons treated for 10 min with SMase (1 unit/ml) or an equal volume of vehicle (control), as well as responses from cells that were pretreated for 15 min with NOE (150 μM). Right panel, bar chart of the charge transfer during the first 10 s of sucrose stimulation showing a 2.5-fold increase in SMase-treated neurons (control, n = 7; SMase, n = 10; ^∗^p < 0.05) and no increase in NOE pretreated neurons (NOE, n = 11; NOE and SMase, n = 7). In the bar charts, error bars represent SEM. The Student's t test was used for pairwise comparisons.

The signaling action of diffusible lipids depends on their release from the membrane environment through enzyme actions. Endogenous sphingosine can be released from membranes through the sequential action of sphingomyelinases and ceramidases (Figure 5A): sphingomyelinase produces apolar ceramide which can flip-flop within lipid bilayer (Contreras et al., 2005) and be hydrolyzed by intracellular ceramidases resulting in the release of amphiphilic sphingosine into the cytosol (Hannun and Obeid, 2008; Lahiri and Futerman, 2007; Van Meer et al., 2008). We first probed the effect of sphingomyelinase action on calcium-dependent glutamate release from isolated rat brain nerve endings. Preincubation of synaptosomes with sphingomyelinase for 10 min followed by addition of 35 mM potassium led to a robust enhancement of Ca^2+^-dependent glutamate release (Figure 5B). Importantly, botulinum neurotoxin D, which proteolyses vesicular synaptobrevin, abolished sphingomyelinase-enhanced glutamate release supporting the conclusion that synaptobrevin is necessary for the sphingosine-mediated enhancement of exocytosis (Figure 5B).

A recent study demonstrated that a mutation of a ceramidase in the *Drosophila* model organism leads to a significant decrease of neurotransmission (Rohrbough et al., 2004) prompting us to employ a ceramidase inhibitor, N-oleoylethanolamine (NOE) (Strelow et al., 2000). Rat brain synaptosomes were preincubated with 150 μM NOE before addition of sphingomyelinase and potassium-triggered depolarization. Figure 5B shows that the ceramidase inhibitor was able to prevent sphingomyelinase-driven enhancement of calcium-dependent glutamate release from the bulk population of central nerve terminals. Next, we assessed action of sphingomyelinase on sucrose-evoked exocytosis which reflects the readily releasable pool of synaptic vesicles (Rosenmund and Stevens, 1996). Addition of sphingomyelinase to cultured mouse hippocampal neurons led to a significant enhancement of hypertonic sucrose-induced transmitter release whereas pre-incubation with the ceramidase inhibitor, NOE, blocked the sphingomyelinase effect (Figure 5C). Together, these data show that enzymatically-produced endogenous sphingosine (or its metabolite) has a stimulating effect on synaptic vesicle exocytosis.

### Effects of Sphingosine Derivatives on Activation of Synaptobrevin and Exocytosis

**Figure 6:**
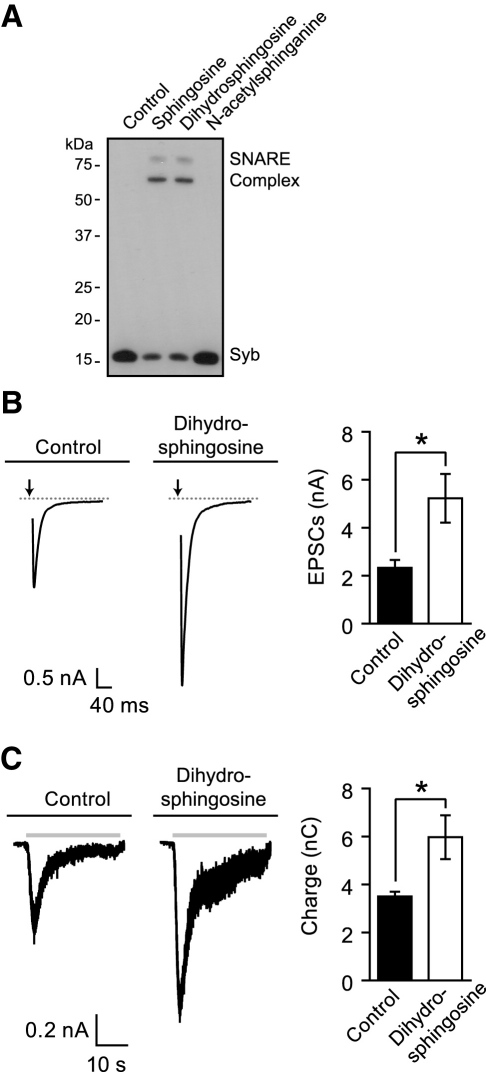
Specific Actions of Dihydrosphingosine, a Sphingosine Mimetic, in SNARE Assembly and in Neurotransmitter Release Experiments (A) Rat brain synaptic vesicles (1 μg protein) were mixed with soluble syntaxin/SNAP-25 in the presence of indicated lipids (50 μM) and analyzed as in Figure 1. SNARE assembly takes place in the presence of sphingosine and dihydrosphingosine, but not in the presence of N-acetylsphinganine. (B) Dihydrosphingosine addition potentiates excitatory neurotransmitter release evoked by field potentials in hippocampal neurons. Left panel, representative traces of EPSCs evoked by field stimulation in wild-type rat hippocampal neurons following treatment with 50 μM dihydrosphingosine or control (DMSO). Arrows indicate the application of current. Right panel, a plot of the average maximum EPSC amplitudes depicting a significant increase in the amplitudes from neurons treated with dihydrosphingosine compared to nontreated neurons (Control, n = 5, Dihydrosphingosine, n = 5; ^∗^p < 0.03). (C) Dihydrosphingosine addition potentiates excitatory neurotransmitter release evoked by hypertonic sucrose (gray bars) in hippocampal neurons. Left panel, representative traces of hypertonic sucrose responses from neurons incubated with either DMSO or dihydrosphingosine. Right panel, bar chart of the charge transfer during the first 10 s of sucrose stimulation showing an increase in dihydrosphingosine-treated neurons compared to control neurons (Control, n = 5, Dihydrosphingosine, n = 5; ^∗^p < 0.03). In the bar charts, error bars represent SEM. The Student's t test was used for pair-wise comparisons.

Since sphingosine can be metabolized in neurons into sphingosine-1-phosphate or back into ceramide, it was necessary to analyze sphingosine analogs which cannot be modified in vivo. For testing, we chose two chemical derivatives—dihydrosphingosine and N-acetylsphinganine, the former being positive regulator of synaptobrevin and the latter being ineffective in our synaptic vesicle assay (Table 1 and Figures 6A and 6B). Addition of dihydrosphingosine to neurons resulted in an increase in both average maximum amplitudes of field potential-stimulated EPSCs (Figure 6B; 2.2-fold) and the hypertonic sucrose responses (Figure 6C; 1.7-fold) compared to neurons treated with vehicle only. In contrast, incubation of neurons with 50 μM N-acetylsphinganine resulted in no significant differences in either EPSC amplitudes or hypertonic sucrose responses (Figure S3). Thus, neurotransmitter release remains unchanged in the presence of N-acetylsphinganine, an ineffective synaptobrevin modulator. In contrast, dihydrosphingosine can both enhance synaptobrevin activity in SNARE reactions and cause potentiation of excitatory neurotransmitter release.

### Mechanism of Sphingosine Action on Vesicular Synaptobrevin

**Figure 7:**
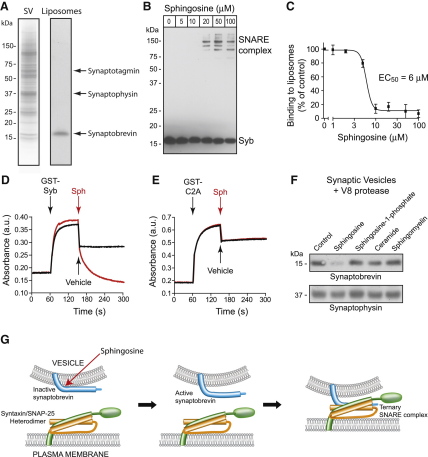
Sphingosine Inhibits Interaction of the Cytoplasmic Part of Synaptobrevin with Vesicular Membranes (A) Protein profiles of purified synaptic vesicles (SV; 30 μg protein) and synaptobrevin liposomes (2 μg protein) visualized in a Coomassie-stained SDS-PAGE gel. Synaptotagmin, synaptophysin and synaptobrevin are prominent synaptic vesicle markers. (B) Liposomes containing synaptobrevin (0.1 μg) were incubated in the presence of soluble syntaxin/SNAP-25 heterodimer (0.3 μg) and increasing concentrations of sphingosine. Immunoblot shows the transition of monomeric synaptobrevin into the SNARE complex. Note that a proportion of reconstituted synaptobrevin resides in the liposomal interior (not shown) and therefore is not available for syntaxin/SNAP-25 binding. (C) The cytoplasmic domain of synaptobrevin (aa 1–96), immobilized on glutathione beads via a GST tag, exhibits robust binding of phosphatidylcholine/phosphatidylserine liposomes labeled by the fluorescent dye DiO. Sphingosine reduces the ability of the cytoplasmic part of synaptobrevin to bind the phospholipid membrane with an EC_50_ of 6 μM. Error bars represent SEM, n = 5. (D) Graph showing increase in absorbance of liposomal solution at 350 nm upon addition of 2.5 μM GST-synaptobrevin (GST-Syb). This increase is immediately reversed upon addition of sphingosine (Sph, 50 μM, red curve). (E) 2.5 μM GST-C2A domain of synaptotagmin-1 induces an increase in absorbance of liposome solution in the presence of 1 mM free calcium. This effect is insensitive to the addition of 50 μM sphingosine (Sph). Vertical drops in absorbance in (D) and (E) are due to dilution of reactions. (F) Limited proteolysis of synaptic vesicles by V8 protease uncovers sensitivity of synaptobrevin, but not of synaptophysin, to sphingosine, as assessed by immunoblotting. Sphingosine-1-phosphate, ceramide, or sphingomyelin (all 50 μM) did not significantly affect proteolysis of the synaptic vesicle proteins. (G) Schematic showing sphingosine-mediated relief of the cytoplasmic part of synaptobrevin from inhibition by the vesicular membrane, a step necessary for further interaction with the syntaxin/SNAP-25 heterodimer. Ternary SNARE complex formation leads to vesicle fusion with the plasma membrane.

Since synaptobrevin resides in synaptic vesicle membrane among a dense network of proteins (Takamori et al., 2006), we probed whether sphingosine acts on the synaptobrevin/membrane interface, or targets synaptobrevin interaction with other vesicular proteins. We reconstituted full-length recombinant synaptobrevin into phospholipid liposomes containing phosphatidylcholine and phosphatidylserine (3:1 molar ratio). Figure 7A shows Coomassie-stained protein content of pure synaptic vesicles and reconstituted proteoliposomes; in the latter case, synaptobrevin is distributed equally in the outer and inner leaflets of liposomal membrane as assessed by trypsinolysis (Hu et al., 2002). Similar to vesicular synaptobrevin, liposomal synaptobrevin was inactive for interaction with syntaxin and SNAP-25 (Figure 7B), demonstrating that phospholipid membrane is sufficient for synaptobrevin restriction. Revealingly, sphingosine was sufficient to overcome synaptobrevin restriction in liposomes when added in a similar concentration range to that required for activation of synaptobrevin in synaptic vesicles (Figures 7B and 1C). In contrast, when we incorporated syntaxin/SNAP-25 into liposomes and tested SNARE assembly in the presence of a soluble part of synaptobrevin, sphingosine did not enhance the ability of this membrane-free synaptobrevin to form SNARE complex with syntaxin and SNAP-25 (Figure S4). Thus, membrane-embedded syntaxin/SNAP-25 heterodimers are available for SNARE assembly and sphingosine appears to affect specifically synaptobrevin in lipid membranes.

Several studies demonstrated the ability of the cytosolic part of synaptobrevin (amino acids 1–96) to directly interact with negatively charged phospholipid membranes (Caccin et al., 2003; de Haro et al., 2004; Quetglas et al., 2000). We analyzed whether sphingosine could compromise the ability of the cytoplasmic domain of synaptobrevin (aa 1–96) to bind fluorescently-labeled phospholipid liposomes in a standard pull-down procedure (Davletov and Sudhof, 1993). Binding of liposomes to synaptobrevin 1–96 immobilized on beads was significant as assessed by bound fluorescence (Figure 7C). When the reactions were repeated in the presence of sphingosine, the liposomal binding to immobilized synaptobrevin 1–96 was severely compromised with an EC_50_ of 6 μM (Figure 7C). To probe whether sphingosine can affect a preexisting synaptobrevin/membrane interaction, we followed membrane binding by GST-synaptobrevin (aa 1–96) in real time using a spectrophotometric assay. This assay detects crosslinking of liposomes by lipid-binding proteins which are dimerized by the GST tag (Connell et al., 2008). GST-synaptobrevin 1–96 was first injected into liposomal solution followed by addition of a sphingosine-containing solution and the two-step reaction was continuously recorded. Addition of the synaptobrevin construct led to an immediate increase in membrane crosslinking (Figure 7D). Sphingosine at 50 μM concentration efficiently reversed liposomal cross-linking (Figure 7D) demonstrating that it can act even after the onset of synaptobrevin/phospholipid interaction. We also tested the well-documented binding of the synaptotagmin C2A domain to the liposomes (Connell et al., 2008; Davletov and Sudhof, 1993). This interaction occurs strictly in a calcium-dependent manner and plays a role in calcium-dependent triggering synaptic vesicle exocytosis (Fernandez-Chacon et al., 2001). Figure 7E shows that sphingosine was ineffective in disrupting synaptotagmin/membrane binding, highlighting a specific mode of action of sphingosine on vesicular synaptobrevin and also indicating that sphingosine does not compromise integrity of phospholipid liposomes. Together, our biochemical results suggest that sphingosine activates vesicular synaptobrevin for SNARE assembly by liberating its cytosolic part from a restriction by the resident phospholipid membranes.

To confirm the ability of sphingosine to affect synaptobrevin properties on the membranes, we performed a limited proteolysis experiment. We noticed that the V8 protease at 0.1 μM concentration cannot readily digest synaptobrevin in synaptic vesicles. Addition of sphingosine allowed almost complete proteolysis of synaptobrevin but not of a control protein, synaptophysin (Figure 7F). In contrast, neither sphingomyelin, ceramide nor sphingosine-1-phosphate were able to promote synaptobrevin digestion. This result confirms that, in the sphingomyelin pathway, sphingosine is the key metabolite that affects biochemical properties of vesicular synaptobrevin. Unlike other sphingomyelin metabolites, sphingosine carries a positive charge at physiological pH due to its free amino group (pKa 8.5; Bottega et al., 1989). To better understand the mechanism of action of sphingosine on vesicular synaptobrevin, we analyzed the requirement of specific groups using ten related compounds (natural and synthetic). Two unique characteristics of sphingosine were identified to be important for synaptobrevin activation—the positive charge and the length of the carbon tail. Indeed, both acetylation of the amino group and phosphorylation of the terminal hydroxyl are sufficient to neutralize the positive charge making ceramides and sphingosine-1-phosphate inactive toward vesicular synaptobrevin (Figure S5). Short C12-sphingosine was also inactive demonstrating importance of the long carbon tail of sphingosine. The fact that L-sphingosine was as effective as the natural D-stereoisoform (Figure S5) demonstrates that the activation of synaptobrevin is not due to a “lock-and-key” mechanism but rather involves a perturbation of the local synaptobrevin environment.

## Discussion

A number of recent studies highlighted the physiological significance of sphingolipid metabolism in vesicle exocytosis. Sphingomyelinase activity has been linked with neurotransmitter release from cerebellar and mesencephalic neurons and also PC12 cells (Blochl and Sirrenberg, 1996; Jeon et al., 2005; Numakawa et al., 2003). A recent genetic study has provided evidence for direct involvement of a ceramidase in synaptic transmission (Rohrbough et al., 2004). Furthermore, a downstream sphingosine metabolite was shown to upregulate glutamate secretion in primary hippocampal neurons (Kajimoto et al., 2007) and spontaneous acetylcholine release at the frog neuromuscular junction (Brailoiu et al., 2002). While ceramide and sphingosine-1-phosphate have been investigated extensively, there is little information about potential ability of sphingosine to regulate vesicle exocytosis (Colombaioni and Garcia-Gil, 2004). Through the screen of lipid compounds, we determined that sphingosine can potently and with high specificity activate vesicular synaptobrevin to form the ternary SNARE complex, an essential step in vesicle fusion. We also showed that sphingosine can enhance vesicle exocytosis in neuromuscular junctions, hippocampal neurons, brain synaptosomes and neuroendocrine cells. Both synaptobrevin-knockout and botulinum neurotoxin experiments (Figures 4 and 5B) indicated that synaptobrevin is necessary for the sphingosine-mediated enhancement of exocytosis.

Our results suggest that sphingosine can relief membrane-mediated inhibition of the cytoplasmic part of synaptobrevin, thereby allowing formation of the ternary SNARE complex. It is conceivable that the positively charged sphingosine can locally disrupt electrostatic and hydrophobic interactions of the cytoplasmic part of synaptobrevin with vesicular membranes. Of note, the cytoplasmic part of synaptobrevin carries basic amino acids centered around hydrophobic tryptophans, all of which can contribute to its interaction with negatively charged phospholipid membranes (Kweon et al., 2003; Quetglas et al., 2000). Recently a consensus has emerged that even in simplified liposomal systems SNARE-mediated fusion requires certain activating mechanisms (Mahal et al., 2002; Tucker et al., 2004; but see Siddiqui et al., 2007). Addition of an artificially truncated synaptotagmin or removal of phosphatidylserine from synaptobrevin liposomes was necessary to stimulate calcium-dependent SNARE-mediated liposomal fusion (Stein et al., 2007; Tucker et al., 2004). We now show that a naturally-occurring signaling molecule, sphingosine, can both accelerate synaptic exocytosis and enhance SNARE assembly.

Preparation of vesicles for fusion with the plasma membrane requires a series of steps: tethering, docking and priming. It is well established that phospholipase C-mediated diacylglycerol production plays an important role in priming of syntaxin for SNARE-mediated vesicle fusion (Wierda et al., 2007). Our data suggest that sphingosine can act as a priming factor for vesicular synaptobrevin at the stage of SNARE complex assembly preceding the calcium-triggered steps. This conclusion is supported by our biochemical data and by the ability of sphingosine to increase the frequency of MEPPs in neuromuscular junctions and sucrose-mediated neuronal exocytosis (Figures 3 and 4B). Our results do not rule out additional effects of sphingosine in synaptic vesicle exocytosis, for example, a change in biophysical properties of the vesicular and plasma membranes. It is well established that sphingosine concentrations are tightly regulated inside the cell (Hannun and Obeid, 2008; Lahiri and Futerman, 2007). Bulk concentration of free sphingosine in mouse brain was estimated to be 0.5 μM (Dasgupta et al., 2007) whereas estimates in rat pituitary cells gave a value of ∼5 μM (Blom et al., 2006). A recent study demonstrated the presence of sphingosine kinase in synapses arguing that production of sphingosine and its further phosphorylation must take place in these secretory compartments (Kajimoto et al., 2007). Since the Michaelis constant for sphingosine kinase was estimated to be ∼15 μM (Pitson et al., 2000), it is likely that such sphingosine concentration can be achieved even if in a local setting. Both synaptic vesicles and the plasma membrane carry sphingomyelin and ceramide (Lahiri and Futerman, 2007; Takamori et al., 2006), and it has been estimated that a single vesicle contains approx. 500 sphingomyelin molecules (Takamori et al., 2006). Pertinently, experiments with sphingomyelinase demonstrated that sufficient amounts of sphingosine can be released from neuronal membranes for activation of both SNARE assembly and exocytosis (Figure 5B and S2).

In summary, our study defines a new mechanism for synaptic regulation: increased sphingosine production results in activation of synaptobrevin for SNARE complex assembly thereby facilitating synaptic transmission. We demonstrated that the unique properties of sphingosine are required for relieving the amphipathic cytoplasmic part of synaptobrevin from its association with the phospholipid membrane. This step is followed by synaptobrevin engagement of syntaxin/SNAP-25 resulting in SNARE assembly and an augmented pool of readily-releasable vesicles. Such a mechanism could explain, for example, stimulation of neurotransmitter release by growth factors that activate sphingomyelin hydrolysis (Blochl and Sirrenberg, 1996; Numakawa et al., 2003). Importantly, it was recently shown that the levels of sphingomyelin and its metabolites are altered in the brain of patients with Alzheimer's disease, Nieman-Pick's disease, and other neurological conditions (Dasgupta et al., 2007; He et al., 2008; Matsuda et al., 2007; Rodriguez-Lafrasse and Vanier, 1999) raising the possibility that deregulation of synaptobrevin function takes place in these disorders. The identification of synaptobrevin as a molecular target of sphingosine may also have implications for current development of drugs which act as sphingosine mimetics, for example, for the treatment of multiple sclerosis (Kappos et al., 2006).

To gain a better understanding of sphingosine signaling in subcellular settings, it will be useful to develop fluorescent reporters that can sense local release of this lipid metabolite similarly to a recent utilization of a protein domain in defining sites of diacylglycerol production (Oancea et al., 1998). Further studies are also required to characterize signaling pathways governing sphingolipid metabolism in neurons allowing a better understanding of synaptobrevin regulation. The discovery of synaptobrevin as a molecular target for sphingosine does not rule out further targets in the complex array of molecules involved in vesicle exocytosis but rather provides a new argument for exploration of the role of lipids in vesicle fusion.

## Experimental Procedures

### Purification of SNARE Proteins

Plasmids encoding glutathione *S*-transferase (GST) fusion proteins of rat syntaxin 1A (aa 1–261), synaptobrevin-2 (aa 1–96 and full-length), wild-type SNAP-25B and SNAP-25B devoid of cysteines were described (Connell et al., 2007; Kweon et al., 2003). Recombinant proteins were released from GST by incubating beads with thrombin, and further purified on a Superdex 200 column equilibrated in 100 mM NaCl, 20 mM HEPES, pH 7.3 (buffer A). The full-length synaptobrevin was purified in buffer A containing 0.8% *n*-octylglucoside (Sigma). Brain syntaxin and SNAP-25 were purified as described (Hu et al., 2002). Protein concentrations were estimated using Coomassie Plus reagent (Pierce).

### Lipid Screening

Recombinant syntaxin was mixed with an equimolar ratio of SNAP-25 overnight at 4°C to allow formation of syntaxin/SNAP-25 heterodimers. Synaptic vesicles (1 μg of protein) were incubated with syntaxin1/SNAP-25 (2 μg) in the presence of 50 μM lipids (Biomol) for 30 min at 22°C. Reactions were analyzed by SDS-PAGE followed by immunoblotting using a synaptobrevin antibody (clone 69.1, Synaptic Systems).

### Preparation of Liposomes and Liposome Binding Assays

Liposomes were prepared as described (Connell et al., 2008). The bead binding assay was performed with fluorescent liposomes containing 1% DiO essentially as described (Davletov and Sudhof, 1993). Bound lipid was quantified using a fluorescence plate reader (Safire 2, Tecan). Real-time liposome binding assay was performed as described (Connell et al., 2008). SNARE proteoliposomes with lipid–protein ratio of ∼200:1 mol:mol were prepared as described (Hu et al., 2002).

### Isolation of Synaptic Fractions and Proteolysis of Synaptic Vesicles

Synaptosomes were prepared from rat brains using Ficoll (Sigma-Aldrich) gradient as described (Davletov et al., 1998). To probe diffusion of sphingosine across membranes, 3 ml of synaptosomes (1 mg protein/ml) were incubated with ^3^H-labeled sphingosine (Perkin-Elmer) for 10 min at 37°C and purified again on the Ficoll gradient. Synaptosomes were disrupted by the hypo-osmotic shock (10 mM HEPES; pH 7.3). Synaptic membranes were isolated by centrifugation (20 min; 34,000 × g; 4°C). Synaptic vesicles were purified as described (Hu et al., 2002). Radioactive sphingosine was quantified by liquid scintillation. Electron microscopy was performed as described (Hu et al., 2002). For limited proteolysis, synaptic vesicles (4 μg of protein) were incubated in the presence of V8 protease (0.1 μM, Sigma) and lipids (50 μM) for 20 min at 37°C. Proteolysis was assessed by immunoblotting using synaptobrevin and synaptophysin antibodies (clones 69.1 and 7.2, Synaptic Systems).

### Release of Glutamate from Rat Brain Synaptosomes

Freshly-isolated synaptosomes were resuspended in synaptosomal buffer B containing (in mM) 132 NaCl, 5 KCl, 20 HEPES, 1.2 NaH_2_PO_4_, 1.3 MgCl_2_, 0.15 Na_2_EGTA, 1 MgSO_4_, 5 NaHCO_3_, 10 D-glucose. Synaptosomes (1 mg protein/ml) were incubated for 10 min at 37°C with an equal volume of synaptosomal buffer B containing glutamate dehydrogenase (15 units/ml, Sigma) and 3 mM NADP (Sigma) in the presence of either sphingosine solution or vehicle, DMSO. Glutamate release was induced by addition of KCl (35 mM) in the presence of 2 mM CaCl_2_ and monitored by following fluorescence (McMahon et al., 1992). *S. aureus* sphingomyelinase (1 unit/ml, Sigma) was added to synaptosomes 15 min before addition of Ca^2+^/KCl. N-oleoylethanolamine (150 μM, Sigma) was added to synaptosomes 15 min prior to addition of sphingomyelinase. Six nanomolar botulinum neurotoxin D was added to synaptosomes 30 min prior to further additions.

### Patch-Clamp Experiments in Rat Melanotrophs and Bovine Chromaffin Cells

Animals were sacrificed in accordance with the European Communities Council Directive (86/609/EEC). Melanotroph cell cultures were prepared from rats as described (Rupnik and Zorec, 1992). Cells were patch-clamped and their membrane capacitance (C_m_) was measured at 22°C using the uncompensated whole-cell technique with a dual-phase lock-in patch-clamp amplifier (SWAM IIC, Celica, Slovenia). Melanotrophs were voltage clamped at −70 mV. Whole cell currents were stimulated with lock-in frequency of 1591 Hz and were collected by Cell software (Celica). The recording bath solution consisted of (mM) 130 NaCl, 10 HEPES/NaOH (pH 7.2), 10 glucose, 8 CaCl_2_, 1 MgCl_2_, and 5 KCl. Patch-clamp pipettes had a resistance of 1.5–4 MΩ and were filled with a solution containing (mM): 150 KCl, 2 MgCl_2_, 10 HEPES/KOH (pH 7.2), 2 Na_2_ATP, 2 EGTA, 1.74 CaCl_2_, yielding free [Ca^2+^] of 1 μM. Secretory responses were measured as a change in C_m_ (%) relative to the resting C_m_ determined immediately after the establishment of the whole-cell recording. The average steady-state currents were −12 ± 8 pA (control), −6 ± 16 pA (10 μM sphingosine), and −25 ± 16 pA (50 μM sphingosine); these values were not statistically different (p = 0.7, Student's t test).

In bovine chomaffin cell experiments, Ca^2+^ current peak (*I*_Ca_) was recorded in voltage-clamped cells under whole cell configuration at 25°C. Cells were dialyzed with a solution containing (mM): 10 NaCl, 100 CsCl, 20 TEA-Cl, 0.1 EGTA, 20 HEPES, 5 MgATP, 0.3 NaGTP (pH 7.2). During recordings, cells were perfused with the Tyrode solution containing (mM) 137 NaCl, 1 MgCl_2_, 10 CaCl_2_, 10 glucose, 10 HEPES (pH 7.4). For patching the cells, pipettes of 3–5 MΩ resistance were used. Electrophysiological data were acquired with an EPC-9 amplifier using the Pulse software (HEKA Elektronik). C_m_ changes were estimated by the Lindau-Neher technique. A 400 ms sinusoidal wave (1 kHz, 60 mV peak-to-peak amplitude) was given before the depolarizing protocol followed by a 2 s sinusoidal wave of the same characteristics, allowing calculations of C_m_ changes. Membrane current was sampled at 20 kHz. Cells were held at −80 mV and depolarizing pulses at +10 mV were applied at 1 min intervals. *I*_Ca_ was analyzed after the initial 5 ms of each depolarizing pulse. To eliminate interference from Na^+^ channel gating (Horrigan and Bookman, 1994), exocytic peak (ΔC_m_) was calculated by subtracting the basal mean C_m_ obtained 400 ms prior to depolarization from that obtained 50 ms after the end of the depolarizing pulse. Initial leak currents were not significantly different: −11 ± 5 pA (control), −10 ± 8 pA (10 μM sphingosine), and −9 ± 4 pA (50 μM sphingosine).

### Electrophysiology of Mouse Hippocampal Neurons and Neuromuscular Junctions

Control and *synaptobrevin-2*-deficient dissociated cultures (courtesy of Dr. Thomas C. Südhof) were prepared as described (Schoch et al., 2001). The hippocampus was dissected and dissociated from day 18 embryos, and dissociated cells were plated on zero-thickness 12 mm glass coverslips and stored at 37°C with 5% CO_2_ in a humidified incubator. We used a modified Tyrode's solution that contained (mM): 145 NaCl, 4 KCl, 2 MgCl_2_, 10 glucose, 10 HEPES, 2 CaCl_2_ (pH 7.4). Pyramidal neurons were whole-cell voltage clamped at −70 mV with borosilicate glass electrodes (3–5 MΩ). Electrode solutions contained (in mM) 105 Cs-methanesulphonate, 10 CsCl, 5 NaCl, 10 HEPES, 20 TEA-Cl, 4 Mg-ATP, 0.3 GTP, 0.6 EGTA, 10 QX-314 (pH 7.3, osmolarity 290 mOsM). Hippocampal cultures (10–21 days in vitro) were treated with lipids (50 μM) for 10 min at 22°C, washed thoroughly and then recordings were performed. Field stimulation was achieved by applying 20 mA pulses with a 1 ms duration using a bipolar platinum electrode; excitatory postsynaptic currents (EPSCs) were measured in the presence of 50 μM picrotoxin. Hypertonic sucrose responses were recorded by infusing modified Tyrode's solution containing 500 mM sucrose, 1 μM tetrodotoxin, 50 μM picrotoxin for 30 s. For enzymatic experiments, rat hippocampal cultured neurons were pretreated for 15 min with NOE (150 μM) followed by 10 min with sphingomyelinase (1 unit/ml) before the application of hypertonic sucrose.

Experiments on neuromuscular junctions were carried out using adult mouse diaphragm at 22°C. Muscles were continuously perfused with a solution containing (mM): 130 NaCl, 5 KCl, 2 CaCl_2_, 1 MgCl_2_, 11 glucose, 12 NaHPO_4_, 2.4 NaHCO_3_ (pH 7.3; gassed with 95% O_2_ and 5% CO_2_). Recordings of spontaneous miniature end-plate potentials (MEPPs) were performed using glass microelectrodes as described (Giniatullin et al., 2006). The recorded MEPPs were digitized at 50 kHz and analyzed off-line to calculate mean values of signal frequency and amplitudes. Sphingosine was dissolved in DMSO and then applied to the muscle via the superfusion system (2 ml/min) while sucrose was applied by local fast microperfusion system using a glass pipette.

### Estimation of Intraneuronal SNARE Assembly Using Botulinum Neurotoxins

Following 30 min incubation with botulinum neurotoxins neurons were washed with PBS, and lysed in SDS-containing sample buffer supplemented with 10 mM MgCl_2_ and 100 units/ml benzonase (Novagen). Proteins were assessed by SDS-PAGE of boiled protein samples (10 μg protein) followed by immunoblotting using indicated antibodies.

## Figures and Tables

**Table 1:** Lipids Tested as Potential Activators of Vesicular Synaptobrevin

Lipid Name	Activation	Lipid Name	Activation	Lipid Name	Activation
(±)4-Hydroxynon-2-enal	−	AGC	−	12-Methoxydodecanoic acid	−
Epoxy-oleic acid	−	Farnesylthioacetic acid	−	**Sphingomyelin**	−
Prostaglandin A1	−	25-Hydroxyvitamin D3	−	**D-erythro-Sphingosine**	+
Prostaglandin A2	−	1,25-Dihydroxyvitamin D3	−	**L-erythro-sphingosine**	+
Prostaglandin B1	−	24,25-Dihydroxyvitamin D3	−	**D-erythro-N,N-Dimethylsphingosine**	+
Prostaglandin B2	−	Retinoic acid, all trans	−	**D-erythro-N,N,N-trimethylsphingosine**	+
Prostaglandin E1	−	9-cis Retinoic acid	−	**D-erythro-Dihydrosphingosine**	+
Prostaglandin F1a	−	13-cis Retinoic acid	−	**DL-threo-Dihydrosphingosine**	+
Prostaglandin I2 Na	−	4-Hydroxyphenylretinamide	−	**D-erythro-Sphingosylphosphoryl choline**	+
15-Keto-prostaglandin E2	−	AM-580	−	**D-erythro-Sphingosine-1-phosphate**	−
15-Keto-prostaglandin F2a	−	TTNPB	−	**Dihydro-sphingosine-1-phosphate**	−
13,14-Dihydro-15-keto-prostaglandin F2a	−	Methoprene acid	−	**D-erythro-N-Acetylsphingosine**	−
6-Keto-prostaglandin F1a	−	WY-14643	−	**D-erythro-N-Acetylsphinganine**	−
16,16-Dimethyl-prostaglandin E2	−	Ciglitazone	−	**D-erythro-N-Octanoylsphingosine**	−
U-46619	−	Clofibrate	−	**D-erythro-N-Octanoylsphinganine**	−
9b,11a Prostaglandin F2	−	5,8,11-Eicosatriynoic acid	−	**D-erythro-N-Palmitoylsphingosine**	−
9a,11b Prostaglandin F2	−	5,8,11,14-Eicosatetraynoic acid	−	DL-PDMP	
Prostaglandin J2	−	1,2-Didecanoyl-glycerol (10:0)	−	DL-PPMP	−
Carbacyclin	−	1,2-Dioctanoyl-SN-glycerol	−	MAPP, D-erythro	−
(±)13-Azaprostanoic acid	−	1,2-Dioleoyl-glycerol (18:1)	−	MAPP, L-erythro	−
19(R)-Hydroxy-prostaglandin E2	−	1-Oleoyl-2-acetyl-glycerol	−	PAF C16	−
17-Phenyl-trinor-prostaglandin E2	−	1-Stearoyl-2-arachidonoyl-glycerol	−	Lyso-PAF C16	−
D12-Prostaglandin J2	−	Ricinoleic acid	−	PAF C18	−
13,14-Dihydro-prostaglandin E1	−	1-Hexadecyl-2-arachidonoyl-glycerol	−	PAF C18:1	−
8-epi-Prostaglandin F2a	−	1-Hexadecyl-2-O-methyl-glycerol	−	Enantio-PAF C16	−
15-Deoxy-Δ^12,14^-prostaglandin J2	−	1-Hexadecyl-2-O-acetyl-glycerol	−	Arachidonoyl-PAF	−
Misoprostol, free acid	−	REV-5901	−	2-EPA-PAF	−
Thromboxane B2	−	LY-171883	−	2-DHLA-PAF	−
11-Dehydro-thromboxane B2	−	SQ-29548	−	2-DCHA-PAF	−
Anandamide (20:4, n-6)	−	Fluprostenol	−	1-Hexadecyl-2-methylglycero-3 PC	−
Palmitylethanolamide	−	Cloprostenol Na	−	1-Octadecyl-2-methylglycero-3 PC	−
Anandamide (18:2,n-6)	−	Eicosapentaenoic acid (20:5, n-3)	−	C-PAF	−
Anandamide (20:3,n-6)	−	Gamma-linolenic acid (18:3, n-6)	−	1-Acyl-PAF	−
Anandamide (22:4,n-6)	−	Eicosatrienoic acid (20:3, n-3)	−	Lysophosphatidic acid	−
Mead ethanolamide	−	Dihomo-gamma-linolenic acid	−	L-NASPA	−
(R)-Methanandamide	−	Docosatrienoic acid	−	Phosphatidic acid, dipalmitoyl	−
BML-190	−	Adrenic acid (22:4, n-6)	−	AM-251	−
N-Arachidonoylglycine	−	Docosapentaenoic acid	−	6-Formylindolo [3,2-b] carbazole	−
WIN 55,212-2	−	Arachidonic acid	−	Diindolylmethane	−
Arachidonamide	−	Docosahexaenoic acid	−	N-Linoleoylglycine	−
Linoleamide	−	17-Octadecynoic acid	−	Palmitoyl dopamine	−
9,10-Octadecenoamide	−	2-Hydroxymyristic acid	−	Oleoyl dopamine	−
Acetyl-farnesyl-cysteine	−	2-Fluoropalmitic acid	−	Arachidonoyl dopamine	−
AGGC	−	4-Oxatetradecanoic acid	−		

+ indicates positive regulators. − indicates inactive compounds. Sphingolipids are in bold.

## Supplemental Data

Document S1. Five Figures
