# Supplementary material for: Sphingosine Facilitates SNARE Complex Assembly and Activates Synaptic Vesicle Exocytosis
Source: Neuron. 2009 Jun 11;62(5):683–94. doi: 10.1016/j.neuron.2009.04.024 (PMC2697323; doi:10.1016/j.neuron.2009.04.024)
Supplement: Document S1. Five Figures [file mmc1.pdf]

Neuron, Volume *62*

Supplemental Data

Sphingosine Facilitates SNARE Complex Assembly

and Activates Synaptic Vesicle Exocytosis

Frederic Darios, Catherine Wasser, Anastasia Shakirzyanova, Artur Giniatullin, Kerry Goodman, Jose L. Munoz-Bravo, Jesica Raingo, Jernej Jorgačevski, Marko Kreft, Robert Zorec, Juliana M. Rosa, Luis Gandia, Luis M. Gutiérrez, Thomas Binz, Rashid Giniatullin, Ege T. Kavalali, and Bazbek Davletov

Supplementary Fig. 1:

Internally dialysed sphingosine augments exocytosis in bovine chromaffin cells.

(A,B) Cells were depolarised by a series of sequential 200 ms pulses separated by 60 sec and changes in membrane capacitance were recorded using the patch-clamp technique. Individual traces are shown in A (control, DMSO) and B (50 μM sphingosine). (C) Bar chart showing relative increase in membrane capacitance upon dialysis of 10 and 50 μM in chromaffin cells. Exocytotic peak (ΔC_m) was calculated by subtracting the basal mean C_m obtained 400 ms prior to depolarisation from that obtained 50 ms after the end of the depolarizing pulse. For each pulse, the capacitance value obtained in the absence of sphingosine was taken as 100 % and relative sphingosine-induced increase was determined. The bar chart shows averaged values for 5 sequential pulses. Error bars represent SEM, n = 3.

Supplementary Fig. 2:

Sphingosine- and sphingomyelinase treatments of hippocampal neurons promote SNARE assembly as detected by botulinum neurotoxin (BoNT) proteolysis.

SNARE complex assembly was analysed using a SNARE protection assay where synaptobrevin and SNAP-25, following ternary complex formation, become resistant to botulinum neurotoxin cleavage. Intraneuronal proteolysis of synaptobrevin, or SNAP-25, was assessed following 30 min treatment of hippocampal neurons with 2 nM BoNT type D, or type E, respectively. Addition of sphingosine (50 μM) or sphingomyelinase (1 U/ml), 10 minutes prior to the BoNT treatments, led to protection against the two toxins suggesting increased SNARE assembly. Following SDS-PAGE of boiled protein samples (10 μg/lane), immunoblotting was performed using synaptobrevin and SNAP-25 antibodies.

Supplementary Fig. 3:

Addition of N-acetylsphinganine to hippocampal neurons does not affect the excitatory neurotransmitter release evoked by either field potentials or hypertonic sucrose.

(A) Field potential stimulation: Left panel, representative traces of excitatory postsynaptic currents (EPSCs) evoked by field potential stimulation in wild-type rat hippocampal neurons after a 10 minute treatment with 50 μM N-acetylsphinganine or an equal volume of vehicle (control). Right panel, bar chart of the average maximum EPSC amplitudes from neurons treated with N-acetylsphinganine compared to control neurons. (B) Hypertonic sucrose stimulation: Left panel, representative current traces from control- and N-acetylsphinganine-treated neurons after hypertonic sucrose application. Right panel, bar chart showing the average charge transfer during the first 10 s of the 30 s hypertonic sucrose stimulation of N-acetylsphinganine-treated and control neurons. Error bars represent SEM, n = 5.

Supplementary Fig. 4:

Sphingosine does not affect the ability of membrane-free synaptobrevin to engage syntaxin/SNAP-25 incorporated in liposomes.

Top panel, schematic showing the relative distribution of SNARE proteins in the experiment. Bottom panel, synaptobrevin immunoblot showing SNARE assembly following 30 min incubation of the cytoplasmic part of synaptobrevin (aa 1-96) with liposomes carrying full-length syntaxin and SNAP-25. Total protein concentrations were 0.2 μM and lipid/protein ratio of the syntaxin/SNAP-25 liposomes was ~200:1 (mol:mol).

Supplementary Fig. 5:

Both positive charge and length of the carbon chain of the sphingosine molecule are important for synaptobrevin activation.

Ten compounds (20 μM) with structures related to the sphingosine molecule were tested for the ability to activate vesicular synaptobrevin for SNARE complex formation in a 60 min reaction at 22°C. SNARE assembly was assessed by Western immunoblotting using a synaptobrevin antibody. Critical structural differences between sphingosine and a given compound are highlighted in *red*. C indicates a control reaction in the absence of lipids.

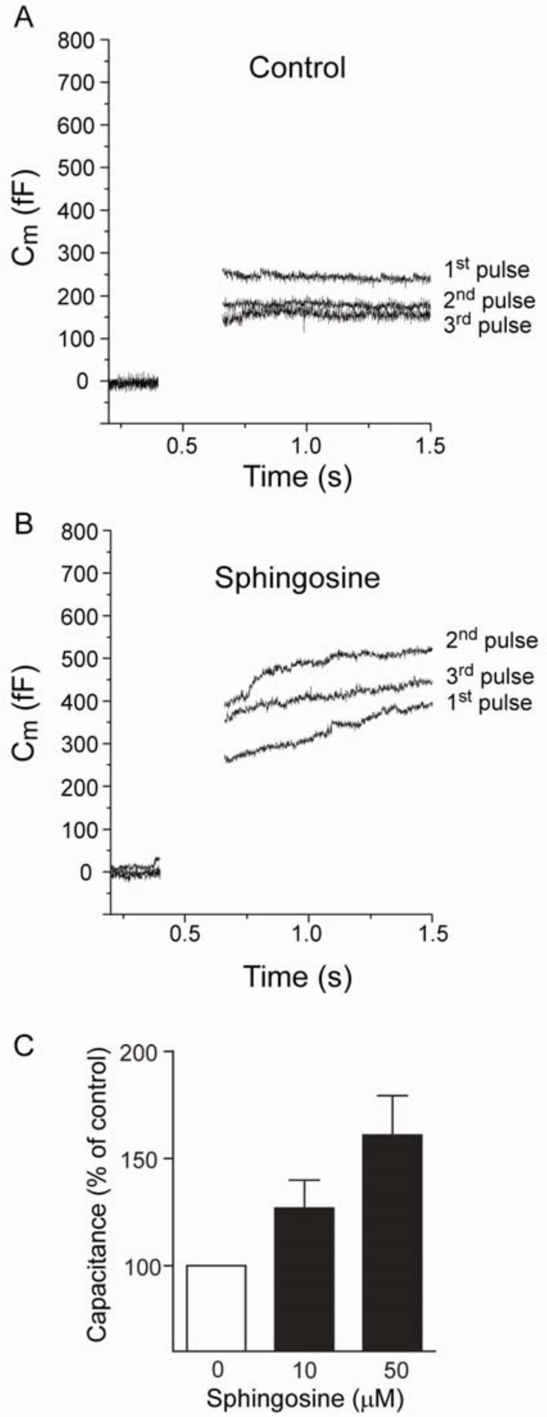

Darios et al, Supplementary Figure 1

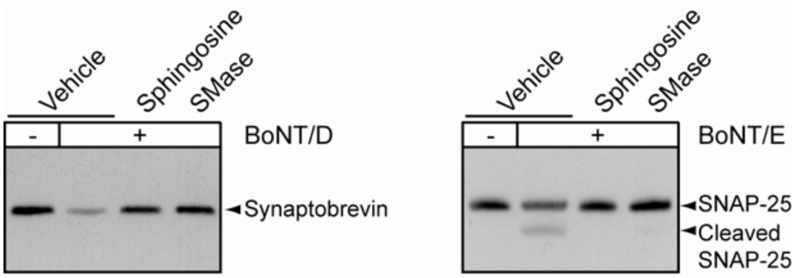

Darios et al, Supplementary Figure 2

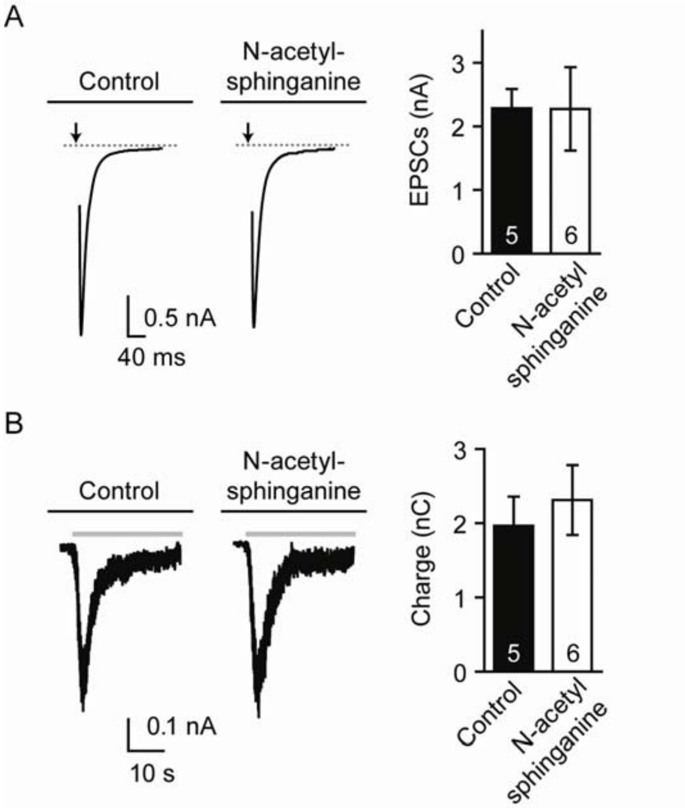

Darios et al, Supplementary Figure 3

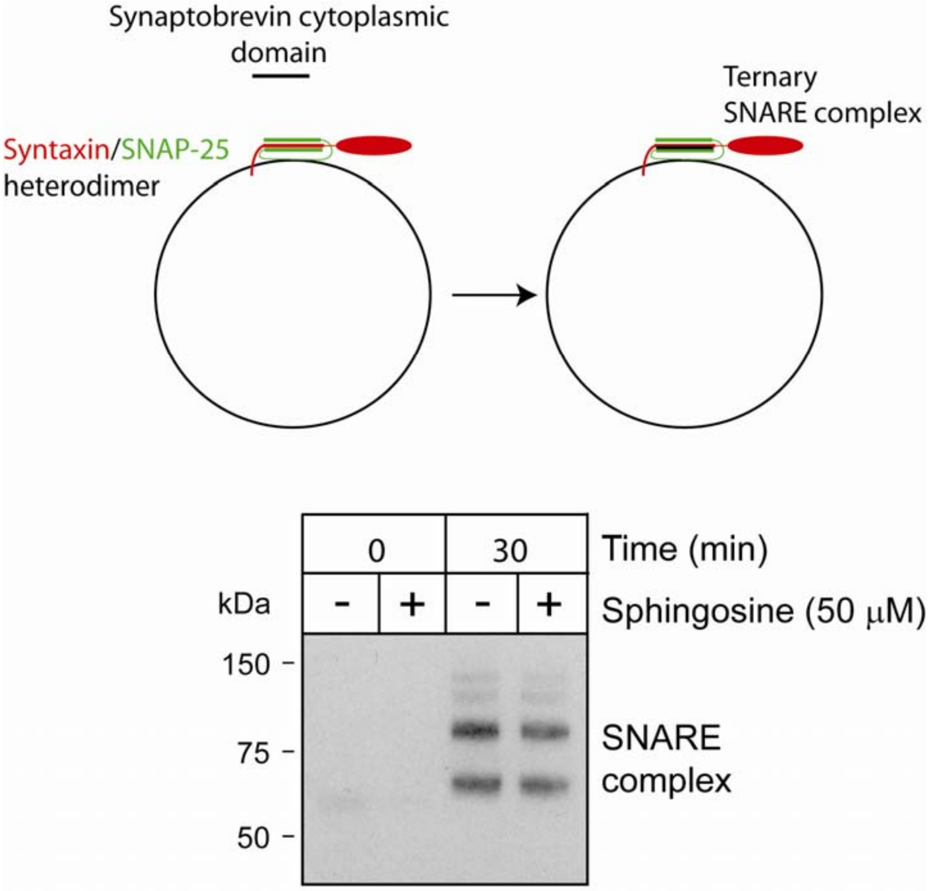

Darios et al, Supplementary Figure 4

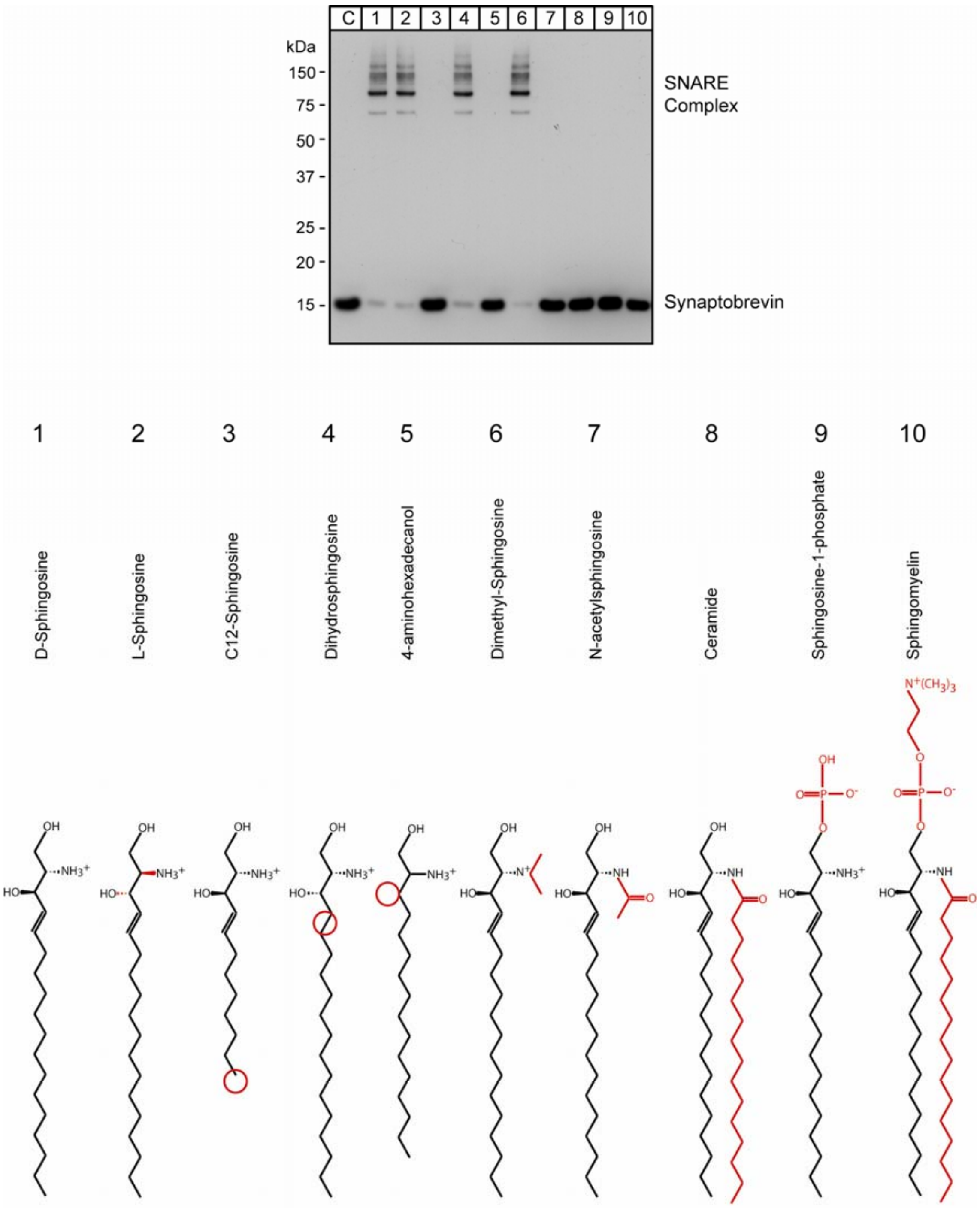

Darios et al, Supplementary Figure 5
